# Supplementary material for: Gut microbiome shifts in adolescents after sleeve gastrectomy with increased oral-associated taxa and pro-inflammatory potential
Source: Gut Microbes. 2025 Feb 19;17(1):2467833. doi: 10.1080/19490976.2025.2467833 (PMC11845021; doi:10.1080/19490976.2025.2467833)

**SuppFig.1.** **Pre-VSG, microbiome diversity and composition related to Type 2 Diabetes status differed, but resolved post-VSG**. (A) Subjects with established diabetes exhibited significantly distinct bacterial microbiota diversity (P < 0.05). (B) Microbiota composition was also highly distinct between subjects with established T2DM and those without (Bray Curtis PERMANOVA R^2^=0.44, P = 0.001). Abbreviations: VSG: vertical sleeve gastrectomy, T2DM: type 2 diabetes mellitus, PERMANOVA: Permutational Analysis of Variance.


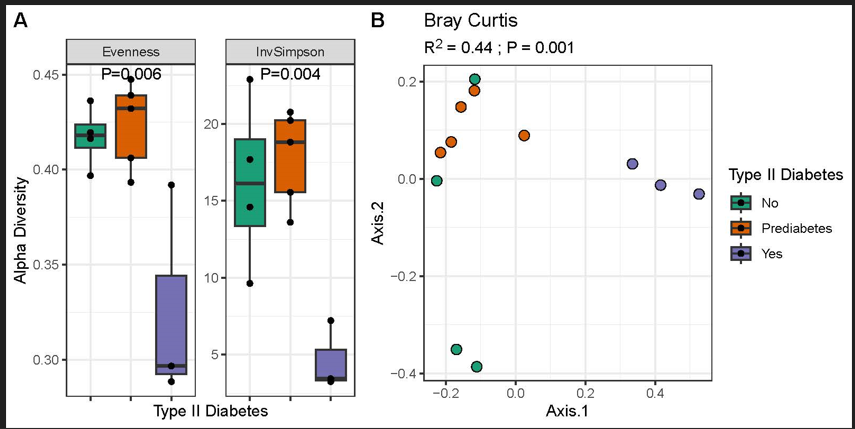


**SuppFig.2. Enzyme Commission genes found to be differentially abundant post-VSG formed three distinct modules of co-associated genes.** (A) PCoA Plot of Enzyme Commission composition using Canberra distance. R^2^ and p-values are derived from PERMANOVA. (B) Differentially abundant enzyme class post-VSG. Enzyme class to the left decrease post-VSG and enzymes to the right increase post-VSG. (C) Hierarchical clustering representation of WGCNA modules based on moderately differential (FDR P < 0.4) pre- and post-VSG. Identified modules are represented by their color underneath the dendrogram. (D) All modules exhibited increases in abundance post-VSG (LME P <0.01). (E) Genera in the microbiome that contribute to each of the three differentially abundant networks described in panels (C) and (D). Abbreviations: VSG: vertical sleeve gastrectomy, FDR: False Discovery Rate, LME: Linear Mixed Effects, PCoA: Principal Coordinates Analysis, PERMANOVA: Permutational Analysis of Variance, WGCNA: Weighted Gene Co-Association Network Analysis.


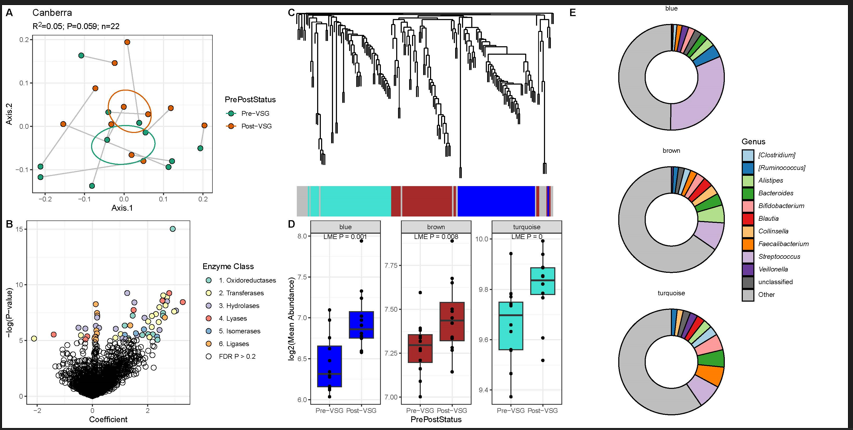


**SuppFig.3. Metagenomic Viral Diversity and composition is not impacted by vertical sleeve gastrectomy.** (A) Identified viruses were primarily comprised of Caudoviricetes. Donut plot represents abundances across both pre-VSG (left) and post-VSG (right) samples for each taxonomic category. (B) Host taxonomy of identified viruses including *Bacteroides*, *Bacteroidaceae*, and *Prevotella,* as well as several with no known hosts. As in (A), plot represents summarized abundances across both pre-VSG (left) and post-VSG (right) samples for each viral host. (C) Viral taxonomic diversity does not change due to VSG with respect to either Chao1 richness or evenness. (D) Viral taxonomic composition does not change signficantly due to VSG. Exploration of either (E) viral Protein Family (PFAM) diversity or (F) composition did not reveal significant differences due to VSG. Points indicate individual samples and lines connect paired samples. P-values for diversity were generated from a linear mixed-effects model with the subject as the random effect. Ellipses in composition plots represent 20% confidence intervals for each group. PERMANOVA calculated the R^2^ and p-values with the subject as the strata variable. Abbreviations: VSG: vertical sleeve gastrectomy, PERMANOVA (Permutational Analysis of Variance), PFAM (Protein Family).


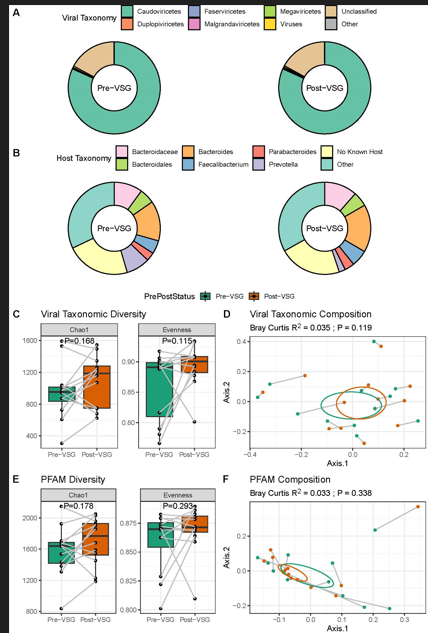


**SuppFig.4.** Metabolites (circles) correlate with clinical variables (squares) (A) prior to and (B) after VSG (p < 0.05). Pink lines indicate positive associations between metabolites and clinical variables while blue lines indicate negative associations. Relationships were identified with non-parametric statistics (Wilcox Rank-sum for binary variables and Spearman correlation for continuous variables). Abbreviations: VSG: vertical sleeve gastrectomy, AA: Amino Acid, FA-Car: Fatty Acid Carnitines, TCA: Tricarboxylic Acid, NT: Neurotransmitter, SCFA: Short Chain Fatty Acid​.


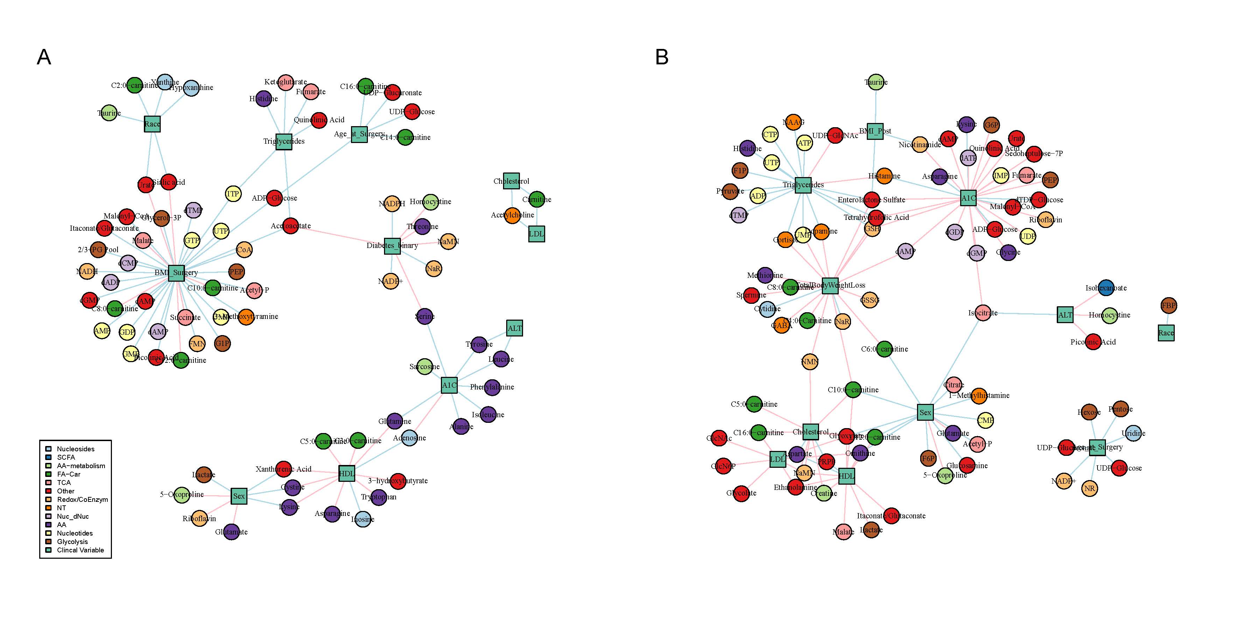


**SuppFig.5**. **Metabolites change in concert with networks of taxa.** (A) Taxa exhibiting at least moderate change after VSG (P FDR < 0.5) formed several co-associated networks using Weighted Gene Co-Association Network Analysis (WGCNA). (B) These networks were characterized by specific taxa that collectively changed in abundance. P-values were generated from linear mixed effects models. (C) These differential networks changed in concert with several changing metabolites. Each line indicates a significant Spearman correlation between the change of metabolites (difference in value before and after) and the change in microbial taxa (difference in mean abundance before and after). Positive correlations are indicated in pink and negative correlations are indicated in blue. The weight of the line indicates the strength of the correlation. Modules identified in panels (A) and (B) are noted by their characteristic taxa. Abbreviations: VSG: vertical sleeve gastrectomy, AA: Amino Acid, FA-Car: Fatty Acid Carnitine, TCA: Tricarboxylic Acid, NT: Neurotransmitter, SCFA: Short Chain Fatty Acid​.


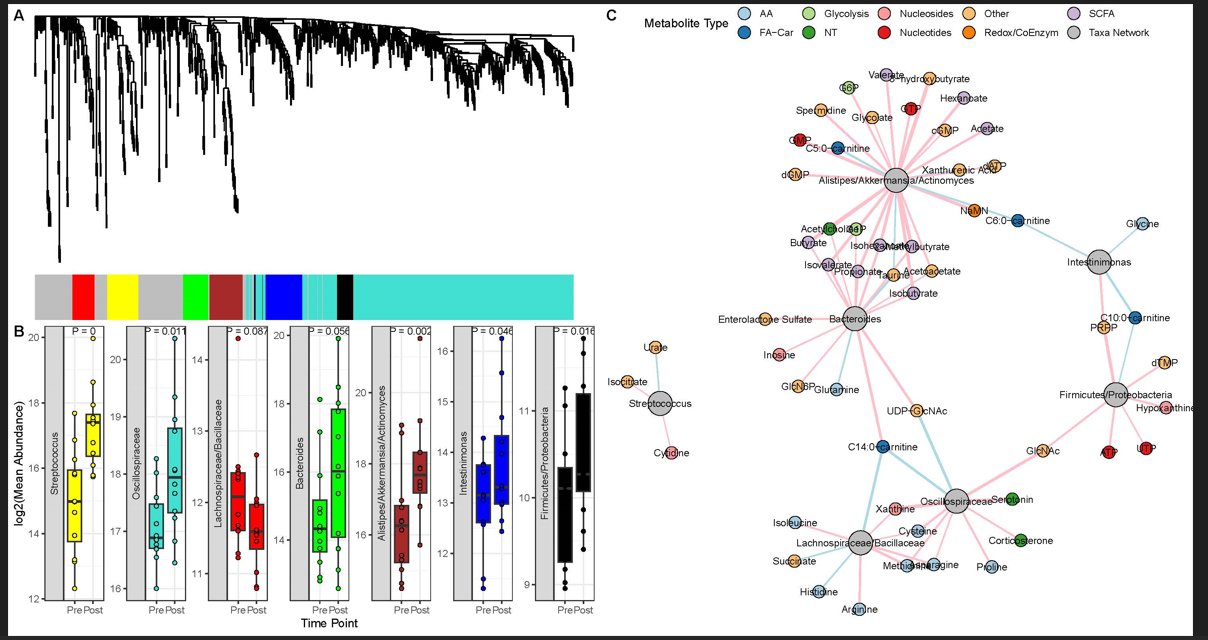


**SuppFig.6. Additional flow cytometry data of mice receiving FT with pre-VSG and post-VSG stool**. (A) Gating strategy for lymphoid cells is shown. (B) Number of live lymphoid cells in the large intestine lamina propria and mesenteric lymph node are plotted. (C-D). Number of live cells for each of the CD4^+^ T cell subsets, their proportion in the mesenteric lymph node (C) and proportion of cytokine producing CD4^+^ T cells (D) are plotted. Groups and participants are identified as shown in the key in (B). Data are displayed as mean ± standard error of the mean. Statistical significance between pre-VSG and post-VSG groups was calculated using unpaired Student’s t-test and non-significance (ns) or p-values are indicated. (E) Top 10 Amplicon sequence variants and flow cytometry features that discriminate pre-VSG vs post-VSG samples from each subject used for FT (n=36 features) identified using mixOmics; rows are z-score scaled. Abbreviations: FT: fecal transplant, VSG: vertical sleeve gastrectomy.


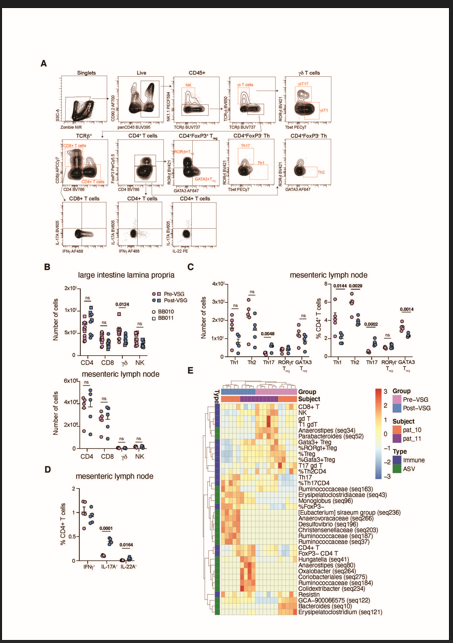


**SuppFig.7. (**A) PCoA plot representing the 6 subjects with increasing calprotectin values post-VSG, in which post-VSG samples become more related to each other. PERMANOVA calculated the interaction between before-vs-after VSG samples (time) and calprotectin. (B) Paired Distance within Pre-VSG samples, within Post-VSG samples, and between Pre-VSG and Post-VSG samples, showing that distance within Pre-VSG samples exhibit higher distance than distances within Post-VSG samples. P-values calculated with Wilcoxon Rank-Sum Test.


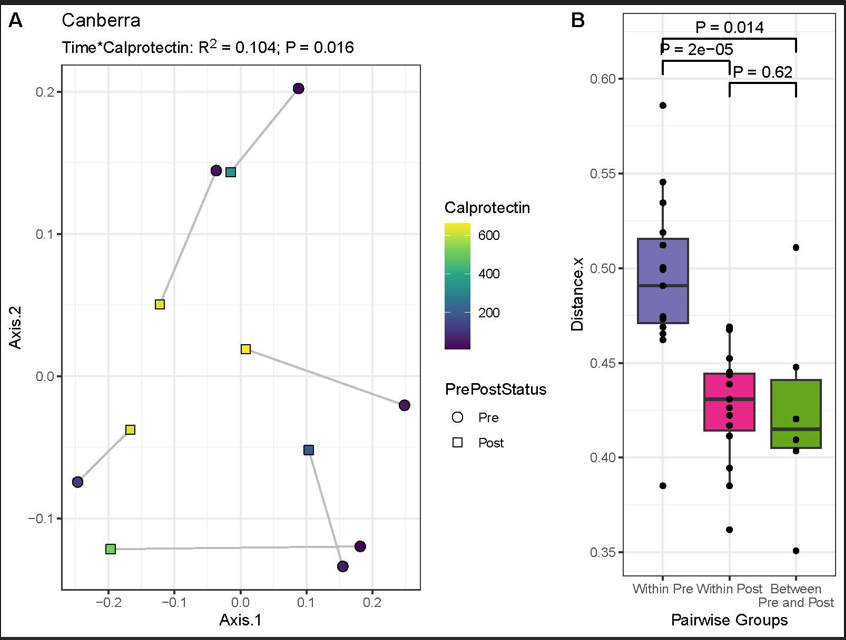


**SuppFig.8.** Validation of urine proteomics against serum proteomics in a separate pediatric cohort. (A) The SomaScan Assay utilizes 7000 SOMAmer Reagents optimized for detection of proteins in peripheral blood serum or plasma. To determine the subset that could be measured in urine, SOMAmer Reagents were classified by comparing their average signal in a cohort of 53 urine samples to background and saturation thresholds. SOMAmer Reagents with a signal-to-noise ratio <1.7 (n=5705) or a saturating signal of RFU >80000 (n=35) were excluded. 1856 SOMAmer Reagents remained within the detectable range of the assay when using urine in this cohort of samples. (B) The samples shown included urine from IBD cases (n=11) and controls (n=20). Comparing these using Wilcoxin Mann-Whitney tests 9 proteins were detected which differed significantly at FDR <0.1. (C) For these 9 proteins that differed significantly between IBD cases and controls in urine, inter-individual variation across the 31 cases and controls was assessed for correlations between the protein levels detected in urine (y-axis) and serum (x-axis).


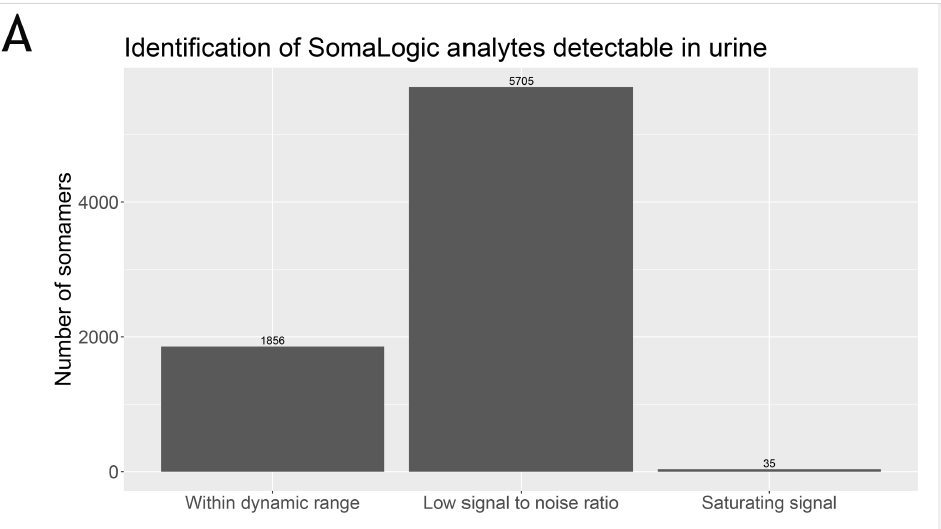

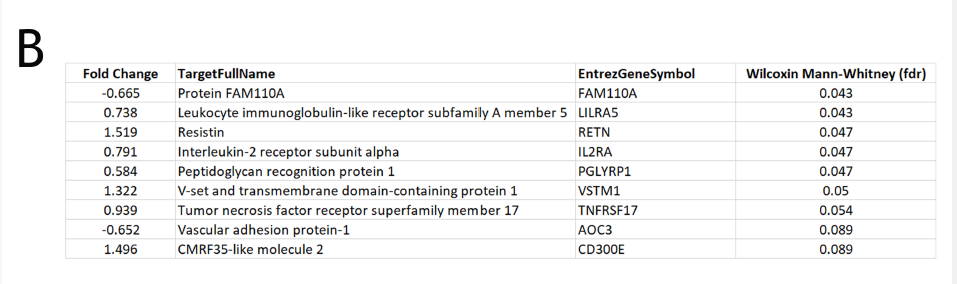


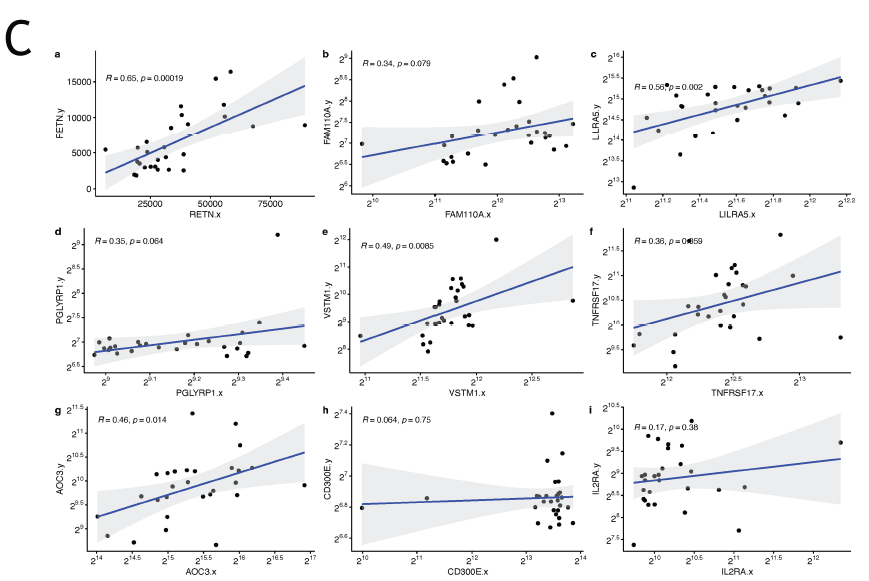

Supplement: Supplemental Material [file KGMI_A_2467833_SM4830.zip › SuppFig_KGMI_A_2467833.docx]
